# Giant plasma cells with multiple immature nuclei in a young woman with newly diagnosed multiple myeloma

**DOI:** 10.1002/jcla.24782

**Published:** 2022-11-17

**Authors:** Valentina Giudice, Danilo De Novellis, Roberto Guariglia, Rosalba Fumo, Giuseppe Ciancia, Bianca Serio, Carmine Selleri

**Affiliations:** ^1^ Hematology and Transplant Center University Hospital “San Giovanni di Dio e Ruggi d'Aragona” Salerno Italy; ^2^ Department of Medicine, Surgery, and Dentistry University of Salerno Baronissi Italy; ^3^ Anatomy Pathology Unit University Hospital “San Giovanni di Dio e Ruggi d'Aragona” Salerno Italy

**Keywords:** multiple myeloma, multinucleated plasma cells, prognosis

## Abstract

**Background:**

Multiple myeloma with giant multinucleated plasma cells is a very rare entity and mostly reported cases are dated. This plasma cell morphology has been observed after monoclonal antibody treatments, such as daratumumab, and patients have experienced a worse prognosis with partial responses and a high rate of relapse.

**Results:**

Here, we showed a newly diagnosed multiple myeloma with giant plasma cells with multiple (up to 13) immature nuclei who achieved a complete remission after a first line therapy and underwent to autologous hematopoietic stem cell transplantation, as per international guidelines.

## Graphical abstract

A newly diagnosed multiple myeloma with giant plasma cells with multiple (up to 13) with loose chromatin, inconspicuous nucleoli, intense cytoplasmatic basophilia, expanded Golgi apparatus, and membrane blebs mimicking a hairy cell leukemia variant. The patient achieved a complete remission after a first line therapy and underwent to tandem autologous hematopoietic stem cell transplantation.

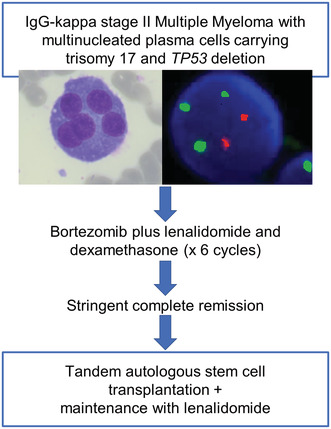

## 1 INTRODUCTION

Multiple myeloma (MM), a clonal malignant hematological condition arising from neoplastic transformation of a plasma cell (PC), is characterized by bone marrow (BM) accumulation of neoplastic clones with hyperproduction of monoclonal immunoglobulins (M‐proteins) detected in serum and/or urine.^1^ PC morphology might vary from mature cells to plasmablasts, or other pleiomorphic forms, such as hairy cell‐like phenotype^2^; however, findings of giant multinucleated PCs are rarely described and poorly investigated in clinical features and outcomes.^3^, ^4^ Here, we report a case of a young female with newly diagnosed MM characterized by multinucleated PCs in the BM and poor prognostic cytogenetic abnormalities successfully treated with induction therapy followed by autologous hematopoietic stem cell transplantation (HSCT).

**FIGURE 1:**
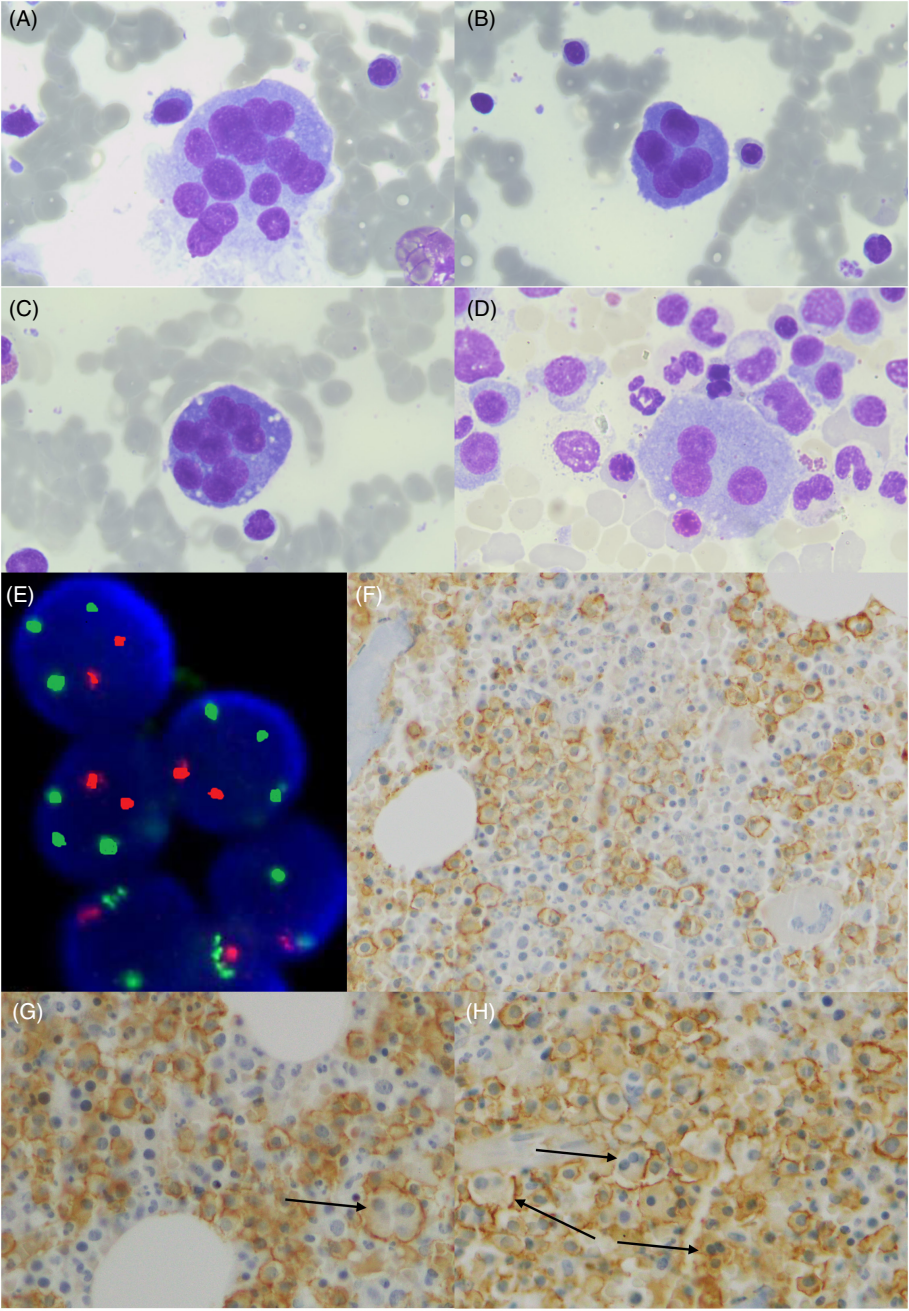
(A–D) Bone marrow blood smears showed giant plasma cells with multiple immature nuclei (from 3 to more than 10), inconsistent nucleoli, extensive Golgi, vacuoles, and cell membrane fragility (Wright‐Giemsa stain, 60× magnification). (E) Fluorescence in situ hybridization analysis for Cep17/p53 green/orange dual‐color probe showing trisomy 17 and *TP53* deletion. (F–H) Bone marrow biopsy immunohistochemistry showed a diffuse CD138+ plasma cell infiltration with multinucleated CD138+ cells (arrows) (40× magnification).

A 46‐year‐old female with a ten‐year history of monoclonal gammopathy of uncertain significance arrived at our observation in November 2017, and an initial evaluation was performed. BM aspirate displayed the presence of 4% of CD38++CD138++CD19‐CD56‐CD45‐ PCs with normal morphology that remained stable until June 2021 re‐evaluation. At that assessment, total BM CD38+ CD138++CD19−/+CD56−/+CD45dim PCs were 17% of total BM mononucleated cells by flow cytometry immunophenotyping, while by light microscopy accounted for 70% of total BM cells. Moreover, around 10% of BM PCs were multinucleated (from three to 13 stratified nuclei), with loose chromatin, inconspicuous nucleoli, intense cytoplasmic basophilia, expanded Golgi apparatus, and membrane blebs mimicking a hairy cell leukemia variant (Figure 1).^2^ Residual CD34+ hematopoietic stem cells (HSCs, 0.6% of total cells) were normally represented by myeloid (97%) and lymphoid (3%) progenitors; however, granulocyte maturation curve showed abnormalities, as neutrophils were mostly CD16‐dimCD11bdim+ intermediate (57%) and CD16+ CD11b+ mature (40%) forms. Serum levels of M‐protein were 2.3 g/dL, β‐2 microglobulin 2.2 mg/dL, albumin 4.1 g/dL, free light chain (FLC) ratio 1.3, and there were no end‐organ damage signs, including hypercalcemia, renal dysfunction, anemia, or bone involvement (CRAB criteria). Trisomy of chromosome 17 and *TP53* deletion were detected by fluorescence in situ hybridization (FISH), identifying a high‐risk cytogenetic profile; however, no abnormalities were found by karyotype analysis. No somatic mutations in *TP53* were identified by next‐generation sequencing, while *CEBPA* (Pro189del; variant allele frequency [VAF], 5.7%) and *SETBP1* (Pro1130Thr; VAF, 48.9%) mutations were detected. Therefore, the patient received a diagnosis of IgG‐kappa stage II MM according to Revised International Staging System (R‐ISS)^1^ in July 2021. Bortezomib plus lenalidomide and dexamethasone (VRD regimen) were employed as induction therapy. The patient quickly achieved a stringent hematologic complete response (sCR) with negative BM minimal residual disease (MRD) by flow cytometry and FISH analysis after just 6 cycles of treatment, and she received the first autologous HSCT in February 2022 and the second in July 2022, according to high‐risk MM treatment guidelines.^1^ At the time of writing after 17 months from the diagnosis of MM, the patient is still in stringent CR and is on maintenance therapy with lenalidomide.

BM giant PCs with more than three nuclei are rare, and their presence has been related to poor outcomes.^3^, ^4^, ^5^ However, revision of 61 BM MM aspirates has revealed that multinucleated PCs can be commonly found (56% of cases) while not influencing clinical outcomes and renal dysfunction development.^6^ In other reports, giant multinucleated PCs have been described in aggressive diseases that only partially responded to chemotherapy, including lenalidomide 25 mg daily and dexamethasone 20 mg weekly, melphalan, prednisone, vincristine, BCNU, adriamycin, or bortezomib and dexamethasone.^3^, ^5^ In some of those cases, multinucleated PCs are detected after anti‐CD20 or anti‐CD38 monoclonal antibody therapies.^2^, ^4^ In our case, giant PCs were found in newly diagnosed MM evolved from a 10‐year preleukemic monoclonal gammopathy. Moreover, the patient obtained a brilliant and deep response with negative MRD after VRD‐based induction therapy consolidated with tandem autologous HSCT as per international guidelines for high‐risk MM.^1^ However, longer follow‐up is needed to precisely estimate the duration of response and progression‐free survival, as giant multinucleated PCs have been also associated with increased relapse rate.^2^

In contrast with reported literature, PCs in our case simultaneously harbored alterations on chromosome 17 and *TP53* deletion with elevated genomic instability. In particular, p53 inefficiency could increase the rate of multinucleated cells because of impaired mitotic processes, as reported in a case of PC leukemia with giant multinucleated cells carrying *TP53* deletion and t(4;14).^7^ However, *TP53* loss in certain nuclei within a scenario of chromosomal mosaicism might be compensated by residual p53 activity in normal nuclei of a multinucleated plasma cell, as in our case. Moreover, since several reports about giant multinucleated MM PCs have been described before the introduction of FISH and molecular biology analysis, *TP53* status is often not reported in previously published cases.^2^, ^3^, ^4^, ^5^ Therefore, we could only speculate on a possible relationship between multinucleated PC presence and high genomic instability that might favor responsiveness to conventional chemotherapy.

## AUTHOR CONTRIBUTIONS

V.G. and C.S. involved in conceptualization; V.G., D.D.N., and B.S. involved in clinical data; R.F. and G.C. involved in molecular and immunohistochemistry analysis; D.D.N. and V.G. involved in writing—original draft preparation; C.S. involved in writing—review and editing. All authors have read and agreed to the published version of the article.

## FUNDING INFORMATION

This research received no external funding.

## CONFLICT OF INTEREST

The authors declare no conflict of interest.

## INFORMED CONSENT

Patients received informed consent obtained in accordance with the Declaration of Helsinki (World Medical Association 2013) and protocols approved by the local ethics committee (Ethics Committee “Campania Sud,” Brusciano, Naples, Italy; prot./SCCE n. 24,988).

## Data Availability

Data are available upon request by the Authors
